# Appendicoliths in Children: Diagnostic Considerations and Postoperative Implications

**DOI:** 10.7759/cureus.90271

**Published:** 2025-08-17

**Authors:** Kiran Mahadevappa, J Asha, Rajkiran S Raju, Deepa Susan John, Prasanna Kumar

**Affiliations:** 1 Pediatric Surgery, St. John's Medical College Hospital, Bangalore, IND; 2 Radiology, St. John's Medical College Hospital, Bangalore, IND

**Keywords:** appendicitis, appendicolith, laparoscopic appendectomy, pediatric, “post-operative complications”

## Abstract

Background and objective

Appendicoliths are calcified deposits within the appendix and are often the cause of complicated appendicitis in children. Their presence is associated with appendicular perforation, abscess formation, and the need for immediate surgical intervention. Preoperative and intraoperative detection influence surgical decision-making and outcomes. This study aimed to evaluate the diagnostic accuracy of imaging, surgical management, and postoperative outcomes associated with appendicoliths in children with acute appendicitis.

Methods

We conducted a retrospective chart review of 198 children aged <18 years who were admitted to the Department of Pediatric Surgery and underwent appendectomy. The data included demographics, imaging findings on ultrasonography (USG) and CT, intraoperative findings, and postoperative outcomes.

Results

A total of 198 children were enrolled in this study. Laparoscopic appendectomy (LA) was performed in 83.3% (165) of the patients, with a laparoscopic-to-open conversion rate of 6.1% (12). Appendicoliths were identified in 15.2% (n=30) of cases on preoperative USG and in 44.9% (n=89) intraoperatively. In a comparison between children with appendicoliths (n=89) and those without (n=109), the presence of appendicoliths was significantly associated with higher rates of perforation (74.2% vs. 14.7%, p<0.001), abscess formation (91% vs. 17.4%, p=0.001), and postoperative wound infection (33.7% vs. 9.2%, p=0.001).

Conclusions

Appendicoliths significantly predispose children to complicated appendicitis. The sensitivity of USG in identifying appendicoliths is limited, and intraoperative detection is associated with a higher incidence of complications than preoperative detection. LA is an effective and safe management option.

## Introduction

Acute appendicitis is one of the most common causes of surgical abdominal emergencies in children worldwide, with an average incidence of approximately 7-12% in the pediatric population [1]. Appendicoliths comprise inorganic elements of calcium and phosphorus, and fecal material, and they are present in approximately 2-4% of the general population, often coincidentally detected on imaging studies. The local appendiceal environment and systemic factors, such as low-fiber diets and constipation, contribute to the formation of appendicoliths [2]. The pathogenesis involves luminal obstruction, bacterial overgrowth in the obstructed lumen, mucosal ischemia, and bacterial translocation, leading to inflammation and the potential for perforation, abscess formation, and gangrene [3]. Children with appendicoliths often have a significantly higher risk of perforation, up to 78% [4,5]; it can be much higher for younger children and infants since they often find it difficult to articulate their symptoms [6].

Prompt and judicious decisions regarding surgical urgency and the suitability of nonoperative management (NOM) are essential for favorable outcomes [7]. The detection of appendicoliths on preoperative imaging is vital for surgical planning, as it may indicate severe disease that requires surgical intervention. However, the sensitivity of ultrasonography (USG) in detecting appendicoliths varies, often leading to underdiagnosis. Incidental appendicoliths are reported in children imaged for unrelated clinical conditions; however, some of these children may progress to appendicitis on follow-up [8]. Incidental appendicoliths have been reported in 2.6% of pediatric CT studies, although the prevalence of asymptomatic appendicoliths in the general population is estimated to be 3% [9]. Only 5.8% of patients diagnosed on CT with appendicoliths develop subsequent appendicitis; its presence indicates a potentially increased risk of developing complicated appendicitis with NOM [7]. Rollins et al. have noted that children with incidental appendicoliths are at a low risk of developing appendicitis and that appendicoliths are often a transient finding [9]. This study aimed to analyze the incidence of appendicoliths in children suffering from appendicitis, their correlation with USG, intraoperative findings, postoperative outcomes, and associated complications.

## Materials and methods

Study setting and ethical approval

This retrospective study was conducted in the Department of Pediatric Surgery over five years (2019-2024) at our institution. The ethical approval was obtained from the Institutional Review Board (IEC) (letter no. IEC/SJMC/Study No. 147 / 2025).

Inclusion and exclusion criteria

The Inclusion criteria were as follows: children aged less than 18 years who underwent appendectomy for acute appendicitis. The exclusion criteria were as follows: patients with a diagnosis of appendicitis managed conservatively, incomplete records, and alternative diagnoses.

Data collection

Demographic data (age and sex), duration of symptoms, imaging characteristics (USG/CT findings, with special reference to appendicoliths), and intraoperative notes were collated and analyzed. Postoperative notes were analyzed with respect to the presence or absence of appendicoliths. Postoperative outcomes included complications (intra-abdominal abscesses, wound infections, and length of stay) and mortality rates. USG was performed by a qualified radiologist in the Department of Radiology, and CT was used selectively when USG was not confirmatory or alternative diagnoses were considered. Appendicolith detection on USG was based on echogenic foci with posterior acoustic shadowing, and that on CT by the presence of hyperdense foci in the appendix. A laparoscopic appendectomy (LA) was performed using a three-port transumbilical approach. Appendix/fecolith retrieval was performed via umbilical (5 mm/10 mm) port. Open appendectomy (OA) was reserved for children with generalized peritonitis, intestinal obstruction, shock persisting despite resuscitation, a history of prior laparotomy with anticipated dense adhesions, or intraoperative challenges such as extensive adhesions, poor visualization, or uncontrolled bleeding.

Statistical analysis

Data were analyzed using STATA software (StataCorp LLC., College Station, TX). All categorical data are presented using frequencies and percentages, and all continuous data are described using the mean and standard deviation (SD) or median and interquartile range (IQR) based on the distribution. The Shapiro-Wilk test was used to assess normal distribution. Complete‑case univariate and multivariate binary logistic regression was used to estimate crude and Adjusted odds ratios (aORs) for factors associated with the Visualization of Appendicolith. The p-value was considered significant at a 5% level for all comparisons.

## Results

**Figure 1:**
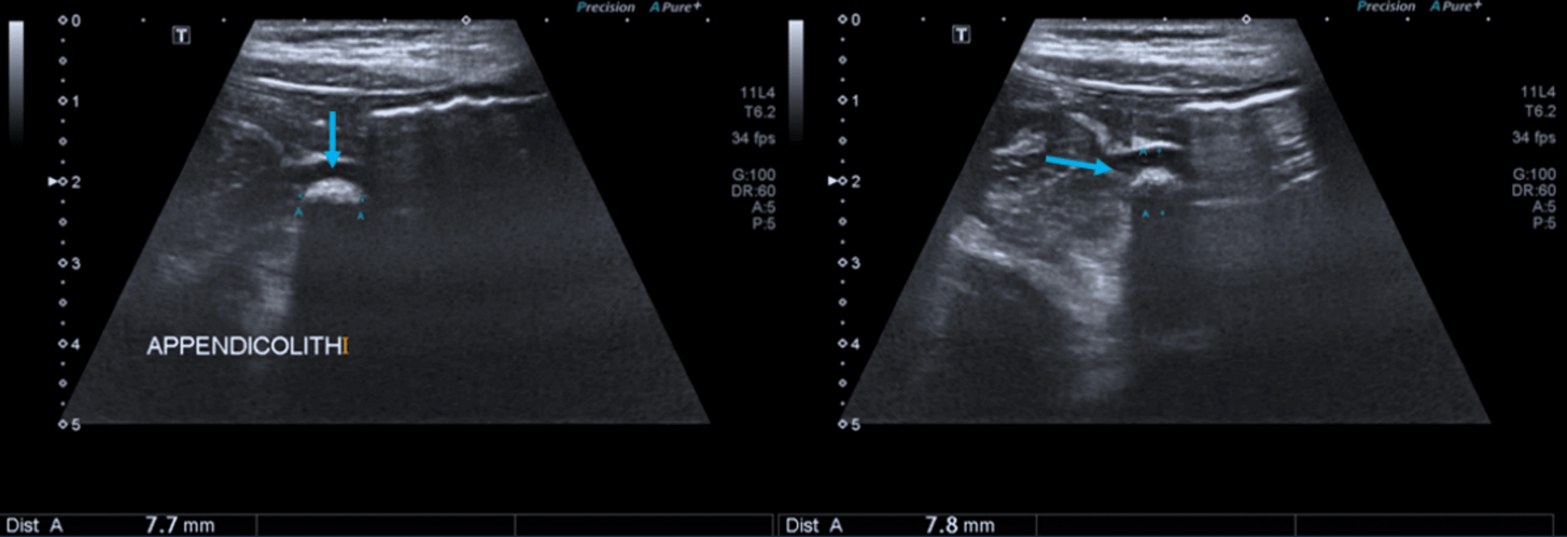
Ultrasound images show an appendicolith with distal acoustic shadowing at the base of the inflamed appendix measuring 7.8 mm

During the study period, 226 patients were diagnosed with appendicitis, and 28 patients were excluded (20 who were managed conservatively, six with incomplete data). A total of 198 children underwent appendectomy. The mean age at presentation was 10.8 ± 3.7 years (range: 2.9-17 years). The cohort was predominantly male (136, 68.7%); the mean duration of symptoms at presentation was 2.3 ± 1.0 days (range: 1-5 days), and 107 patients presented after 48 hours from the onset of symptoms. Preoperative USG detected appendicitis in 66.2% (n=134) of the patients and appendicoliths in 30 (15.2%). The size of the appendicoliths varied between 6 and 15 mm (Figure 1).

**Figure 2:**
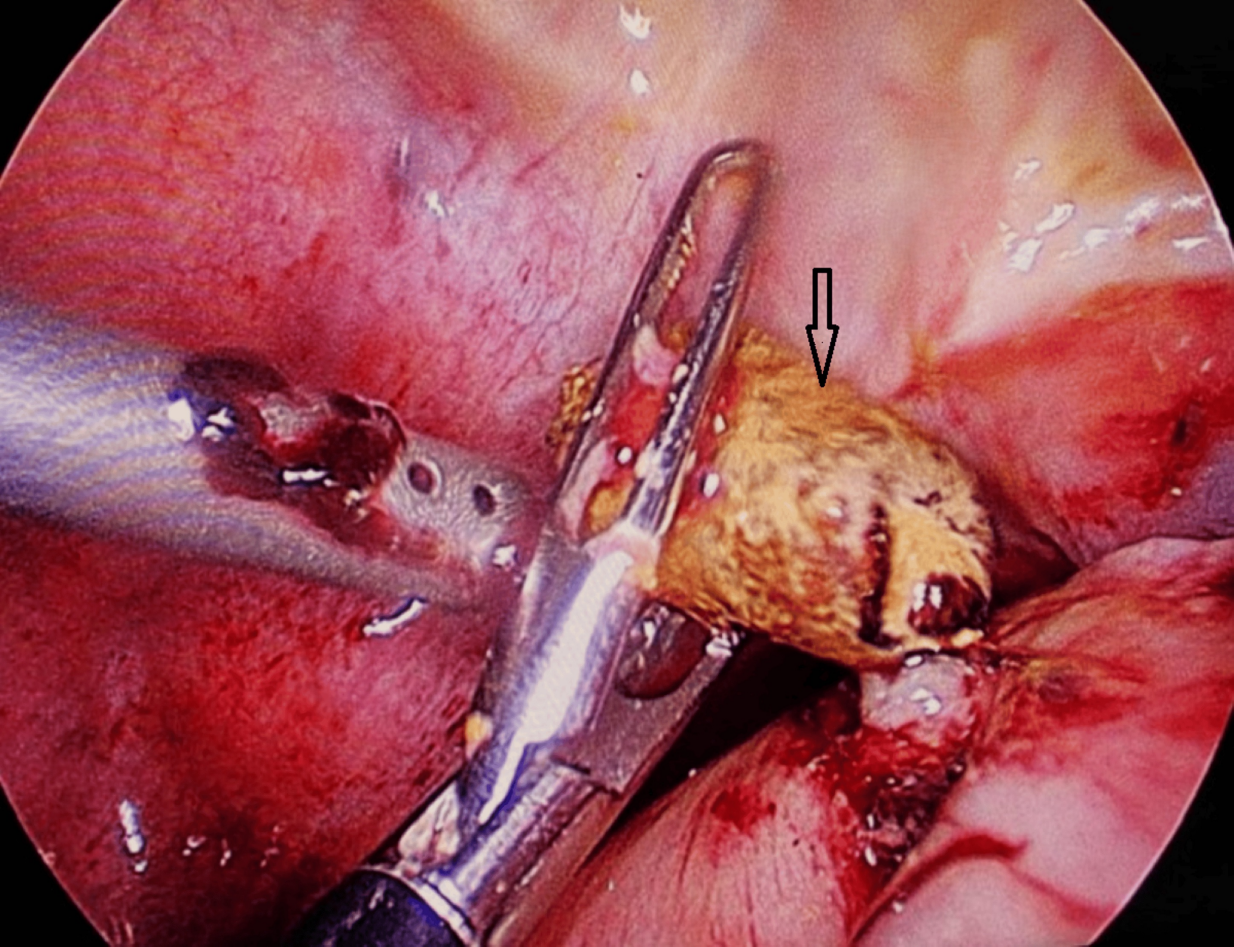
Intraoperative image of appendicolith (laparoscopic)

A CT was performed in 16 (8.1%) patients, and appendicoliths were noted in 13 (81. 3%). Intraoperatively, appendicoliths were observed in 89 cases (44.9%) (Figure 2).

LA was performed in 165 children (83.3%), with 12 (6.1%) requiring conversion to open surgery due to generalized peritonitis (n=7), uncontrolled bleeding (n=1), or dense adhesions (n=4). Primary OA was performed in 21 patients (10.6%) (Table 1).

**Table 1:** Baseline demographic, presentation, and operative characteristics SD: standard deviation; USG: ultrasonography

Variable	Values (N=198)
Age, years, mean ± SD	10.8 ± 3.7
Sex, n (%)	
Male	136 (68.7%)
Female	62 (31.3%)
Presentation to hospital >48 hours after the onset of symptoms, n (%)	
Yes	107 (54.0%)
No	91 (46.0%)
USG, n (%)	
Appendicitis	134 (66.2%)
Appendicolith	30 (15.2%)
Surgical approach, n (%)	
Laparoscopic	165 (83.3%)
Open	21 (10.6%)
Laparoscopic converted to open	12 (6.1%)
Appendicolith visualized intraoperatively, n (%)	
Present	89 (44.9%)
Absent	109 (55.1%)

During the intraoperative period, appendicular perforations were observed in 62 (31.3%) patients and abscess formation in 100 (50.5%) patients. Postoperatively, surgical site infections were identified in 40 (20.2) patients. All the 21 children who underwent primary OA had incisional surgical site infection (SSI); in the Lap converted to open group, 10 children had SSI. In the laparoscopic surgery group, only nine children had port site SSI. The length of hospital stay exceeded five days in 108 (54.5%) patients. None of the patients had a significant postoperative abscess requiring intervention. Immediate postop intestinal adhesions were noted in 8.6 (n=17/198) with ileus; all these patients were managed conservatively without the need for additional surgery. No patient in our series had wound dehiscence or complication requiring surgical re-exploration.

Correlation of appendicoliths with disease severity

The presence of an appendicolith significantly correlated with increased rates of perforation (74.2% vs. 14.7%; p<0.001), abscess formation (91% vs. 17.4%; p<0.001), and wound infection (33.7% vs. 9.2%). Additionally, there was a notable increase in the postoperative length of stay exceeding five days (79.8% vs. 33.9%, respectively).

After multivariable adjustment, only intra-abdominal abscesses and an extended length of hospital stay (exceeding five days) remained significantly and independently associated with the presence of an appendicolith. The initial observed associations with perforation and wound infection are attributable to confounding factors and were no longer evident in the adjusted model (Table 2).

**Table 2:** Univariable and Multivariable logistic regression analysis for factors associated with appendicolith visualization CI: confidence interval

Factors		Appendicolith		Crude odds ratio (95% CI)	P-value	Adjusted odds ratio (95% CI)	P-value
		Visualized (n=89)	Not visualized (n=109)				
Perforation	Yes	46 (74.2%)	16 (14.7%)	6.22 (3.17, 12.20)	<0.001	0.98 (0.35-2.72)	0.971
	No	43 (31.6%)	93 (68.4%)				
Abscess	Yes	81 (91.0%)	19 (17.4%)	47.96 (19.92, 115.50)	<0.001	43.25 (14.57, 128.42)	<0.001
	No	8 (8.2%)	90 (91.8%)				
Wound infection	Yes	30 (33.7%)	10 (9.2%)	5.03 (2.30, 1.04)	<0.001	0.53 (0.18, 1.55)	0.244
	No	59 (76.3%)	99 (62.7%)				
Length of stay >5 days		71 (79.8%)	37 (33.9%)	7.68 (4.00, 14.73)	<0.001	3.01 (1.22, 7.42)	<0.001

## Discussion

The incidence of appendicoliths in children varies among studies, ranging from 32.5% to 54.6% [2,8]. Appendicoliths in pediatric appendicitis are significantly associated with complications such as perforation, abscess formation, and SSI. Decisions regarding surgical treatment, particularly the appropriateness of nonoperative management for complicated acute appendicitis, should be made promptly and carefully. This retrospective case series highlights the significant role of appendicoliths and outcomes of pediatric appendicitis, aligning with the existing literature that emphasizes their importance as markers of complicated appendicitis. 

In the multivariable logistic regression of 198 children (appendicolith visualized in 89, absent in 109), intraoperative appendicoliths were strongly and independently associated only with postoperative intra-abdominal abscesses and prolonged hospitalization. After adjusting for the other complications in the model, the odds of developing an abscess remained exceptionally high (adjusted OR=43.3, 95% CI: 14.6-128.4, p<0.001), and the odds of staying >5 days tripled (adjusted OR=3.0, 95% CI: 1.2-7.4, p<0.001). The crude associations with perforation (unadjusted OR=6.22) and wound infection (unadjusted OR=5.03) were no longer significant after adjustment (adjusted ORs=0.98 and 0.53, respectively; p≥0.24), indicating likely confounders by the co-occurring complications. Thus, the presence of an appendicolith at surgery chiefly signals a markedly increased risk of postoperative abscess and a need for a longer hospital stay, rather than serving as an independent predictor of perforation or wound infection.

Clinical significance

Numerous studies have shown that the presence of appendicoliths is associated with severe complications. Our findings revealed a strong association between the presence of appendicoliths and perforation rates (74.2% vs. 14.7%, p<0.001), aligning with Yoon et al., who found that children with appendicoliths had a perforation rate of 43.5%, which was significantly higher than that in children without appendicoliths. This is supported by Paya et al., who discovered that perforated appendicitis was almost twice as prevalent in patients with appendicoliths (OR=2.27) [10]. Additionally, Yoon et al. pointed out that larger appendicoliths (≥5 mm) and proximal collapse significantly increase the risk of perforation, highlighting the importance of size and location in risk assessment [5].

Imaging

USG is a reliable and affordable diagnostic tool. A meta-analysis of pooled data of 26 studies involving 9356 patients with acute appendicitis demonstrated a sensitivity and specificity of 88% and 94% for ultrasound as compared to 94% and 95%, respectively, for CT evaluation [11]. Ultrasound has been reported to have a high specificity of 98.7% in the diagnosis of acute appendicitis but a very low sensitivity of 54.9% in differentiating simple and complicated appendicitis (complicated appendicitis on USG were periappendicular abscess or a perforated or necrotic appendicitis with localized or generalized pyoperitoneum) [12]. In our study, preoperative ultrasound detected appendicitis in 66.2% with a sensitivity of 66.8%, and appendicoliths were seen in 15.2% of cases with a sensitivity of 33.7%, whereas appendicoliths were noted during surgery in 44.9% of cases. Another large retrospective study of 1027 patients by Gonzalez et al. reported the sensitivity/specificity of ultrasound in the detection of appendicolith to be 58.1% and 78.3%, respectively [13]. This may partly be attributable to the subjective errors arising from operator skill or patient-related factors such as uncooperative patients and obesity [8].

Although USG is safe and economical, it tends to underestimate disease severity. High-resolution CT scans offer superior performance and specificity for appendicitis and can effectively detect appendicoliths. Baykara et al. reported 88% sensitivity and 57% specificity for CT in the diagnosis of appendicitis, and with greater accuracy in diagnosing appendicoliths [14]. In cases where USG results are inconclusive, particularly in symptomatic patients, it is advisable to consider low-dose CT or other advanced imaging modalities to facilitate timely surgical interventions. Surgeons should be mindful of the potential for elevated complication rates, ensure thorough peritoneal cleansing, and tailor postoperative monitoring accordingly. Patel et al. suggested that MRI can serve as a reliable alternative to CT for diagnosing appendicitis with an overall sensitivity of 95% and a specificity of 94.2% [15].

Management and surgical outcomes

The use of LA in children with complicated appendicitis is a topic of debate because of the challenges presented by anatomical distortion and inflammation in this population. However, recent studies have indicated that LA is safe and effective in these patients. It offers advantages such as fewer postoperative complications, shorter hospital stays, and faster recovery times compared to OA [16]. These benefits suggest that LA is a favorable first choice for treating even complicated appendicitis in children, including perforated appendicitis [17]. However, it may require greater surgical expertise and time than OA. In our study, we employed LA as our standard procedure in most of our cases (n=165, 83.3%), with a conversion rate of 6.1% and low morbidity. Postoperative complications included wound infections in 40 (20.2%) of cases and intra-abdominal abscesses in 8.6% (17) of cases. Patients with appendicoliths experienced a higher rate of wound and abscess complications, highlighting the critical need for thorough intraoperative irrigation and careful follow-up [18]. Khan et al. recommended early laparoscopic intervention for complicated appendicitis, emphasizing the benefits of enhanced visualization and abscess drainage; in their study, 4.3% experienced ileus and 2.2% had superficial wound infections [19].

Implications for practice

The identification of appendicoliths requires careful consideration of the above-mentioned factors. In cases where USG results are inconclusive for appendicitis, particularly for symptomatic high-risk patients, it may be advisable to consider low-dose CT or other advanced imaging modalities to facilitate timely surgical interventions. Surgeons should be mindful of the potential for elevated complication rates, ensure thorough peritoneal cleansing, and tailor postoperative monitoring accordingly.

Limitations and future directions

This study has a few limitations. The retrospective, single-center design of this study limits the generalizability of its findings to the general population. Furthermore, the decision to forego routine CT scans to minimize radiation exposure may have led to an underestimation of preoperative appendicolith detection rates. To enhance diagnostic algorithms and accurately evaluate the impact of appendicoliths, prospective multicenter studies employing standardized imaging protocols are required. Additionally, the feasibility of nonoperative management in cases involving appendicoliths requires further investigation, given the increased risk of perforation and abscess formation.

## Conclusions

Appendicoliths are found in one-third of pediatric appendicitis cases and independently double the odds of perforation in appendicitis. USG misses nearly half of appendicoliths; low-dose CT or, occasionally, MRI should be considered when detection alters the management. LA is a safe and effective procedure; however, patients with appendicoliths exhibit higher postoperative morbidity and require closer postoperative surveillance than those without appendicoliths.
